# Royal Hospital for Incurables

**Published:** 1892-08-20

**Authors:** S. Minton-Senhouse

**Affiliations:** Putney


### Editor's Letter-Box.

[Our correspondents are reminded that prolixity is a great bar to publication, and that brevity of style and conciseness of statement greatly facilitate early insertion.]

### ROYAL HOSPITAL FOR INCURABLES.

Sir,—Being one of the Governors invited to the meeting of July 22nd to consider the Report of the Lords' Committee, and not being able to attend, I sent the following letter, to be put into the Chairman's hands before the meeting commenced. My messenger, however, was rather late, and as it was not read, or, so far as I can learn, mentioned, and being naturally much interested in the Home, after devoting many years of my life to it, taking services, attending meetings, and visiting over and over again every room in the house, I should be glad if you could find room for it in some early issue of your paper. I am, yours truly,

S. Minton-Senhouse.

Putney, August 11th, 1892.

Fair Head, Putney, S.W., July 20th, 1892.

Dear Sir,—During the fifteen years of my ministry at Melrose Hall I scrupulously abstained from interfering, except in a few small details, with its secular management, believing that I should do more good in my own vocation by keeping aloof from questions in which there were sure to be strong differences of opinion. But it is no longer possible for me to keep silent. If the hospital is to remain in existence, decisive action of some kind or other must be taken upon the public challenge which has been authoritatively thrown down to us. And being entirely unable to attend the meeting, necessity compels me to state my views in writing, which, considering the exceptional opportunities that have been so long afforded me of observing the inner workings of the institution under varying administrations, I shall not perhaps be thought unreasonable in requesting you to have read aloud. I fully believe that those chiefly concerned in the management have endeavoured to do their best for the general comfort and welfare of the inmates. But still, I have long thought that some changes were desirable. And whether they are so in themselves or not, it would now be obviously impossible for us to go on as we are in face of the adverse decision given against us so strongly, after public investigation, by a Committee of the House of Lords.

In the first place, then, I think that the resident head of the house should be a man, and not a woman. Man is born to rule. And, although in some cases it may be very difficult to find a suitable man, able and willing to take some particular part of authority, and although, of course, some women are vastly more competent for it than others, there must, by the laws of nature, be almost certainly some drawback when they are placed in positions of extensive command, especially if they are set over a number of men.

It has been objected to the suggestion of a resident medical officer being at the head of the establishment, that comparatively few of the patients require constant medical attendance. And perhaps, for that purpose alone, his whole time could scarcely be needed. But to combine the two offices would certainly occupy him fully. The doubt, however, seems to be whether anyone could be found possessing the double qualification who would accept the post. The essential requirement, in my judgment, is to secure a thoroughly satisfactory Governor, whether he is qualified to add the duties of medical officer or not.

This, of course, would involve the appointment of a Matron for subordinate duties, under the control, where necessary, of the Governor.

But what has always, within my knowledge, been the most earnestly and generally desired is the appointment of an independent committee of visiting ladies who, without any power to interfere officially with the management, would be cordially recognised, both by the authorities and the patients as friendly mediators, to gain the confidence of the latter, and encourage them to speak freely whatever is in their minds, that their grievances may be redressed, and their reasonable wishes attended to. Until this is done, nothing will be done. Persons in their helpless condition require severe pressure to make them prefer complaints to, or through, those who are set over them, at the risk of being made to feel their displeasure for it. They think it better to bear the ills they have, especially if they are directly warned against talking of their affairs to "outsiders," and are given plainly to understand that no interference will be tolerated. This is the very sorest point with them.

Pray excuse my ending abruptly, and believe me, dear sir, yours faithfully,

S. Minton-Senhouse.

To the Chairman of the meeting, Royal Hospital for Incurables, Cannon Street Hotel.

